# Self-Reported Hearing Difficulty Versus Audiometric Screening in Younger and Older Smokers and Nonsmokers

**DOI:** 10.4021/jocmr611w

**Published:** 2011-07-26

**Authors:** Ishara Ramkissoon, Margaret Cole

**Affiliations:** a Ishara Ramkissoon, University of South Alabama 5721 Drive N., HAHN 1119 Mobile, AL 36688, USA; b Margaret Cole, Spring Hill College 4000 Dauphin St. Mobile, AL 36608, USA

## Abstract

**Background:**

The high incidence of age-related hearing loss demands accessible, low cost hearing screenings for prevention and hearing health promotion. This study assessed performance of self report (SR) against audiometry, and prevalence of hearing difficulty when screening hearing in middle-aged and younger adults, including smokers and nonsmokers.

**Methods:**

Prospective participants (N = 219) completed a questionnaire providing biographical, health, and smoking information. Their Yes/No responses about hearing or communication difficulty provided data for self-reported hearing loss. Eligible (N = 170) participants received a hearing test including immittance, pure-tone, and speech audiometry. The binaural pure-tone average (PTA) hearing threshold was determined; PTA decibel (dB) level indicated degree (e.g., mild) of hearing loss. All hearing screening data were coded and initially analyzed in an Access database. Statistical analyses based on conditional probability included measures of prevalence, sensitivity, specificity, and predictive value of the SR versus audiometric measures. Participants provided a urine sample for biochemical analysis to confirm smoker/nonsmoker status.

**Results:**

Among all participants (N = 170), overall prevalence of self-reported hearing difficulty (15.9%) was in excellent agreement with measured, mild hearing loss (16.5%). However, factoring in age and smoking revealed that SR was incongruent with audiometry because hearing loss was overestimated by smokers and younger participants and underestimated by middle-aged individuals. The SR question yielded high specificity (80-90%) overall. Specificity was highest in nonsmokers (89-94%) and younger (90-91%) individuals with lower performance in smokers and middle-aged participants. SR sensitivity was high (86-100%) only when the hearing impairment cutoff was > 40 dB (moderate loss) and > 60 dB (severe loss). Sensitivity was highest in smokers (100%), supporting SR for screenings. High negative and low positive predictive value (PPV) occurred in smokers, younger, and middle-aged persons. This study reports new sensitivity and specificity data on self-reported hearing difficulty in smokers (N = 98), younger (N = 80), and middle-aged (N = 90) adults, indicating efficacy of SR as an adult hearing screening measure.

**Conclusions:**

SR was effective as few normal-hearing persons were labeled “hearing-impaired”. However, audiometry should supplement SR to optimize detection of mild hearing loss for at-risk adults. Results may guide community health initiatives for hearing screenings, prevention, and health promotion.

**Keywords:**

Aging; Smoking; Self Report; Health Promotion; Hearing Screening

## Introduction

In considering the effects of a health condition, the World Health Organization (WHO) promotes a holistic model so that disability is viewed in terms of activity limitations and participation restriction for the affected individual. For example, a chronic disability like hearing loss shows effects including a loss of independence and a poorer quality of life in aging adults [1]. Degree of hearing loss (impairment) is usually defined by pure-tone thresholds where 0-25 dB is considered normal, and mild impairment begins at 26 dB [2]. Adult-onset hearing loss is a major health concern because it is ranked third among chronic health conditions in adults 65 years and older [3], with a prevalence of 33% [4]. Aside from this age-related hearing loss in elderly adults, recent research highlighted an earlier onset of communication difficulties among individuals in the fourth and fifth decade of life. According to the American Speech Language Hearing Association (ASHA), 14% of adults aged 45-64 years have a hearing loss [2]. This is the baby boomer generation (born 1946 to 1964) and they have a higher incidence of hearing loss than previous generations, likely due to lifestyle factors such as smoking and noise exposure [5]. Younger adults are also increasingly exposed to noise from using personal music systems (e.g., MP3 players) at high volume. This has contributed to noise-induced hearing loss in 15% of Americans starting at age 19 years [6]. Thus, screening for adult hearing loss is no longer the exclusive concern of elderly adults. In fact, one objective of Healthy People 2020 is to increase the numbers of persons aged 20-69 years who are screened for hearing loss by their primary care provider, using a standardized approach [7]. ASHA also recommends that all adults undergo hearing screening at least once every decade until age 50, and every three years thereafter [2]. These recommendations facilitate hearing screening services for young and middle-aged adults at risk for hearing loss, which will increasingly be done in community clinics by nurses and other trained personnel.

Hearing screening protocols might be impacted by health disparities. The elimination of health disparities among disabled persons is prioritized in Healthy People 2010 [8]. There are disparities in health risk behaviors such as smoking because 40% of adults with hearing loss versus 24% of those with good hearing were current smokers [9]. The positive association between smoking and hearing loss is highlighted among middle-aged and older adults as current smokers were 1.69 times more likely to have a hearing loss than nonsmokers [10, 11]. Current smoking prevalence was 22% in younger (18-44 years), 22% in middle-aged (45-64 years), and 12% in older (65-74 years) adults [12]. These recent data suggest that smoking status should be considered in hearing screening protocols because it is unknown if smoking impacts screening outcomes. This unknown requires further investigation because hearing loss is a top research priority of the U.S. government working on healthcare reform [13].

Adult hearing screening protocols in the U.S. are not easily accessible, universal, or available at low cost [14]. The changing healthcare landscape includes many more community health clinics offering convenient care, primary health, and preventive care including health promotion programs. Thus, community clinics often provide access to hearing health care services [14], with some states like Alabama and Florida offering hearing screenings by trained technicians. Audiologists seldom provide mass hearing screenings due to a shortage in the professional workforce [15]. The bulk of adult hearing screenings, however, are conducted by family nurse practitioners [16]. Screening practices focus on detecting functional and/or physiologic hearing loss using informal or formal procedures. Nurses and physicians typically determine presence of functional hearing loss by questioning patients about their hearing using informal self report (SR) as a preliminary screening tool [17]. Physiologic hearing loss is measured with an audiometer or pure-tone hearing screener (e.g., Audioscope) [18]. While current screening practices do attempt to address the hearing health needs of millions of adults at risk for hearing loss, a NIH-sponsored research working group recommended further research to determine which screening methods have the highest sensitivity and specificity [14]. Past research on hearing screening methods emphasized the older population experiencing age-related hearing loss [19-23]. Variability in the sensitivity and specificity of SR tools were due to methodological differences among these studies. A large-scale study using the “*Hearing Handicap Inventory for the Elderly-Screening*” (HHIE) for self reported hearing loss indicated higher sensitivity and lower specificity among adults aged 48-64 years (baby boomers) compared to older adults aged 65-92 years [22]. The higher sensitivity in middle-aged than older adults is likely related to elderly individuals under-reporting hearing deficits, as typically observed in clinical practice. Of note, the HHIE is a good screening tool for physicians [24] but it was not designed for middle-aged or young adults. Only one known study focused on a middle-aged population: in a telephone survey, prevalence of self reported hearing loss was 49% among working adults 40-64 years [5, 25]. However, there was no comparison of SR (single question) to audiometry among this baby boomer cohort. Furthermore, no known studies have considered the impact of smoking on hearing screening procedures. In fact, the actual number of older smokers is expected to increase as baby boomers age [26]. There are also no known studies comparing SR to pure-tone hearing loss in young adults, an important gap in the knowledge given recent reports of higher prevalence of hearing loss in younger persons [14].

The current study, therefore evaluated adult hearing screening techniques by examining sensitivity and specificity of a single question SR compared with the gold standard, pure-tone audiometry and an alternate gold standard, word recognition score (WRS) in a sample of healthy, middle-aged and younger adults, including smokers and nonsmokers. It was hypothesized that SR sensitivity and specificity would be high in the group of middle-aged adults [5, 25], SR would have fair sensitivity and high specificity in young adults, and SR would reveal high sensitivity and fair specificity in smokers.

## Methods

### Participants

Data collection was completed at two universities, one Midwest and one in the Southeast following Institutional Review Board approval of study materials and test protocols for human subjects. Participant recruitment targeted healthy adults in two age ranges: 19-30 years (young) and 45 years or older (middle-aged); the 30-44 year range was not included for this study. Recruiting occurred via flyers posted in the community and by word of mouth. Over 300 inquiries were received from prospective participants. A total of 219 individuals completed a questionnaire providing biographical, health, and smoking information. Of these, 49 (22%) were not eligible due to age or health reasons. The remaining 170 participants (78%) who reported good overall health with no alcohol or drug dependency, mental illness, or neurological disease were selected for the study.

Of 170 participants, 61 were male. Mean age of the younger group (N = 80) was 24 years (S = 3.9). Two-thirds of the middle-aged group (N = 90) were baby boomers with a mean age of 62 years (S = 9.3). Participants were initially categorized as nonsmoker (N = 98) or smoker (N = 72) based on their responses to items on the questionnaire with later confirmation by biochemical urine testing. Among nonsmokers, 77 reported having never smoked and 21 were past smokers who had quit at least 3 years before. Among smokers, 60% reported moderate-to-heavy smoking (11-20 cigarettes per day), and 15% smokers reported heavy smoking (> 21 cigarettes per day), defined by benchmark guidelines from the Fagerstrom Test for Nicotine Dependence [27].

### Procedures and Instrumentation

Each participants hearing was estimated by screening with a binary classification test (single question) that was included on the questionnaire. Specifically “*Do you have any hearing or communication difficulties?*” had a Yes/No response option, and participants responses provided the data for self reported hearing loss. Participants attended one 30-40 minute hearing testing session in a research laboratory. A certified audiologist or audiology graduate assistant visualized the external ear canals and tympanic membranes with an otoscope, and then measured each participant’s hearing with pure tone air-conduction, immittance, and speech audiometry. Pure tone testing was conducted in a sound treated booth using a standard two-channel audiometer. Hearing thresholds were measured in each ear for frequencies: 250, 500, 1000, 2000, 3000, 4000, 6000, and 8000 Hertz (Hz). Participants were instructed to listen to sounds of different pitch through insert earphones placed in each ear, and indicate when stimuli were heard by pressing a button, hand raising, or saying "yes". Hearing thresholds, defined as the softest level at which a signal is heard 50% of the time, were determined by standard clinical procedures [28]. Speech audiometry included speech recognition threshold testing and WRS, measured with recorded stimuli (CID-W22) presented at 40 dB suprathreshold and reported as percent correct.

Participants provided a urine sample in a standard collection cup while at the research facility. Each sample was dated, labeled, and stored in a refrigerator at 4^o^C. Urine samples were analyzed with high performance gas chromatography at an independent laboratory for presence/absence of nicotine and cotinine. Biochemical laboratory reports were compared to each participants reported smoking behavior with 100% agreement. This confirmation of participant categorization as a smoker or nonsmoker was the sole purpose of the urine sample.

### Data Management and Statistical Analysis

Participants’ responses on the questionnaire were coded and entered into an Access database program [29]. Self reported hearing/communication difficulties (Yes/No responses), and binaural audiometric test results including pure-tone thresholds and WRS were also entered into the database. Raw data were analyzed to calculate Pure Tone Average (PTA, defined as binaural mid-frequency average (1000, 2000, 3000, and 4000 Hz from each ear) following the method of Gomez et al. [30] because it had highest agreement between SR and audiometry. This study defined audiometric hearing loss by WRS less than 88%, and PTA with a dB cutoff > 25 dB (mild), > 40 dB (moderate), and > 60 dB (severe). A total of 106 queries were made within the Access database that yielded individual reports with information such as “number of middle-aged participants”, “number of younger participants who self reported hearing loss”, and “number of smokers who self reported hearing loss and had PTA > 25 dB”.

For the statistical analyses, participants were grouped as middle-aged (N = 90), younger (N = 80), smokers (N = 72), nonsmokers (N = 98), and All (N = 170). Hearing screening techniques were evaluated with statistical measures of prevalence, sensitivity, specificity, and predictive value, determined within the framework of conditional probability [31]. Prevalence of hearing impairment reflected percentage of the sample that had hearing loss. Statistical sensitivity, specificity, and predictive value were determined for the SR screening test compared with audiometry in four conditions of hearing impairment: mild (PTA > 25 dB), moderate (PTA > 40 dB), severe (PTA > 60 dB), and speech (WRS < 88%).

## Results

The primary purpose of this study was to evaluate an adult hearing screening technique, SR considering age and smoking behavior. Prevalence of hearing loss was estimated by SR and measured by audiometry (Table 1). For the full sample of participants (N = 170), prevalence of SR hearing difficulty was 15.9%, and prevalence of hearing impairment assessed by audiometry was 16.5% (mild), 5.9% (moderate), and 1.2% (severe). Considering age and smoking behavior, prevalence of mild hearing impairment was 31.1% (middle-aged), 0.0% (younger), and 16.7% (smokers). The last column in Table 1 (M - E), showing the difference between the actual hearing test and SR indicates the error was minimal (0.6%) for the full sample of participants, that is a close match. Interestingly, however, when age and smoking behavior were considered, results were incongruent in smokers (-5%) and younger participants (-10%) reflecting an overestimation of hearing loss by SR. Error was 10% in middle-aged individuals revealing the highest underestimation of SR hearing loss.

**Table 1:** Prevalence of hearing impairment (%) from SR and pure tone audiometry by participant age and smoking behavior.

Participants	N	Self Report	PTA > 25 dB (mild)	PTA > 40 dB (moderate)	PTA > 60 dB (severe)	M - E
All	170	15.9	16.5	5.9	1.2	0.6
Middle-aged	90	21.1	31.1	11.1	2.2	10.0
Younger	80	10.0	0.0	0.0	0.0	-10.0
Smokers	72	22.2	16.7	4.2	2.8	-5.6
Nonsmokers	98	11.2	16.3	7.1	0.0	5.1

PTA=binaural pure tone average of 1000, 2000, 3000, and 4000 Hz. M - E is the measured (PTA > 25 dB) minus estimated (SR) prevalence of hearing impairment.

Screening characteristics including sensitivity, specificity, positive predictive value (PPV), and negative predictive value (NPV) of SR compared with audiometric techniques (PTA, WRS) are shown in Table 2. Overall, specificity was high (0.80-0.90) in all four comparisons. Specificity was highest in non-smokers than other participants (smokers, young, middle-aged, all) when hearing loss was mild (PTA > 25 dB) or moderate (PTA > 40dB). However, when hearing loss was severe (PTA > 60 dB) or determined by WRS, specificity was highest for younger participants, with non-smokers coming in marginally second. A closer look at specificity revealed that it is 5-13% lower in smokers compared to other participants at PTA > 25 dB and PTA > 40dB. Furthermore, specificity is 5-10% lower in middle-aged individuals and smokers compared to other participants when hearing loss was severe (PTA > 60 dB) or based on WRS.

**Table 2:** Screening performance characteristics (%) for self reported hearing difficulty compared with pure-tone hearing impairment and WRS.

	Sensitivity	Specificity	PPV	NPV
SR vs. HI (PTA > 25 dB)				
Participants: All	46	90	48	89
Middle-aged	46	90	68	78
Younger	*	90	0	100
Smokers	58	85	44	91
Nonsmokers	38	94	55	88
SR vs. HI (PTA > 40 dB)				
Participants: All	90	89	33	99
Middle-aged	90	87	47	99
Younger	*	90	0	100
Smokers	100	81	19	100
Nonsmokers	86	94	55	99
SR vs. HI (PTA > 60 dB)				
Participants: All	100	85	7	100
Middle-aged	100	80	11	100
Younger	*	90	0	100
Smokers	100	80	13	100
Nonsmokers	*	89	0	100
SR vs. WRS (< 88% LE or RE)				
Participants: All	47	85	23	95
Middle-aged	50	80	29	91
Younger	0	91	0	99
Smokers	56	81	28	93
Nonsmokers	33	89	15	96

SR: self reported hearing difficulty; HI: hearing impairment; PTA: binaural pure-tone average of 1000, 2000, 3000, and 4000 Hz; WRS: word recognition score; LE; left ear; RE: right ear; PPV; positive predictive value; NPV: negative predictive value. * indicates calculation not possible due to number of true positives = zero.

Overall, sensitivity was low (33-58%) in two comparisons and high (86-100%) only when hearing loss was moderate or severe. Sensitivity was highest (56-100%) in smokers than all other participants when hearing loss was mild, moderate, or based on WRS. Sensitivity was excellent (100%) and equal among participants when hearing loss was severe.

In general, PPV scores were low across all comparisons of SR to PTA and WRS. However, PPV was modestly high (68%) for middle-aged participants when SR hearing difficulty was compared to mild hearing impairment. NPV was excellent (89-100%) across all comparisons of SR to PTA and WRS, particularly in younger participants. In middle-aged participants, however, NPV was much lower (78%) when SR was compared to PTA > 25dB.

## Discussion

### Prevalence of Hearing Difficulty

The current prevalence (31%) of measured hearing loss (PTA > 25 dB; mild impairment) in middle-aged participants is an excellent match to earlier reports [4]. In contrast, prevalence of measured hearing loss in younger (0%) participants is on the lower end of the range (0.9-15%) previously reported [6, 9]. This might be due to the current participant selection criteria that targeted individuals with the best hearing for an initial investigation. Also, younger participants in this study might have had less noise exposure than previous reports. For smokers, the prevalence of measured hearing loss (16%) appears to be lower than previously reported, however this prevalence is collapsed across age and actually falls in the range previously reported for older (12%), as well as younger and middle aged (22%) individuals [12].

An interesting new finding in this study regarding the difference between measured and estimated prevalence, indicated that younger participants and smokers overestimated self reported hearing loss. No other known studies have reported how younger individuals or smokers perform on SR versus measured hearing screening tests. This self reported hearing loss that was not verified by physiological hearing changes (PTA > 25 dB) might indicate early signs of functional hearing difficulties. For example, noise exposure in younger persons might lead to temporary threshold shift and similarly, smokers might be experiencing poorer speech understanding in noisy or other challenging listening situations [6, 10, 11].

The difference between measured and estimated prevalence (M - E) revealed that middle-aged individuals in this study underestimated (10%) hearing loss by SR. This result was unexpected. It was anticipated that baby boomers would view even slight hearing difficulties more importantly than elderly individuals [22]. Although our middle-aged group were two-thirds baby boomers, their self reported hearing loss did not match measured hearing loss. This underestimation of hearing loss among middle-aged participants might be supported by anecdotal clinical reports that indicate baby boomers do not want to admit they have any hearing difficulty. Nevertheless, the current result is different than three earlier studies where adults overestimated (1%, 6%, 11%) self reported hearing loss [22, 23, 30]. However, methodological differences, like participant age exists between the current and previous studies. In addition, some studies calculated hearing impairment by worse ear [22] or better ear [23] while this study used both ears (binaural) to better reflect participants overall hearing function and because binaural mid-frequency PTA agreed best with SR [30]. The type of question used to determine self reported hearing loss also differed among studies. This might have contributed to observed differences between studies because language like “*Do you have any trouble hearing*” [30] or “*Do you feel you have a hearing loss*” [23] imply different levels of handicap to participants.

### Screening Characteristics

Discussion of screening characteristics for the comparison of SR to WRS is excluded because it revealed the poorest/lowest sensitivity and specificity indicating its lack of suitability as an alternate gold standard in hearing screening tests. The current study evaluated sensitivity, specificity, NPV, and PPV of self reported hearing difficulty from a single question “*Do you have any hearing or communication difficulties?*” in younger and middle-aged adults, including smokers and nonsmokers. The overall findings indicated high specificity and lower sensitivity of the SR measure compared with audiometry (PTA). This is in keeping with the inverse relationship of the two characteristics. It might be addressed in terms of the relative importance of sensitivity and specificity for prevalence of hearing loss and any consequences of errors in the screening process.

#### Specificity

Middle-aged adults in this study revealed higher specificity than sensitivity of the SR measure which is similar to previous findings in older individuals [19, 30]. Moreover, current specificity in middle-aged individuals was higher (90%) than previously reported [19, 22, 23, 30], likely due to differences in research methodology. A second interesting result in middle-aged adults revealed that specificity of SR decreased as degree of hearing impairment increased. While this appears counterintuitive because one might expect more severe hearing loss to be over-reported, it is similar to behavior patterns of aging adults who under-report hearing difficulty in clinical environments. For example, adults aged over 65 years rated their health positively and described their health status as excellent, very good, or good despite having a chronic condition like hearing loss [32]. Less is known about how middle-aged adults respond to questions about health status. From the telephone survey study [25], 49% of baby boomers self reported hearing loss which suggested that the current sample of middle-aged adults would behave similarly and lead to possible high specificity findings. Instead, the result of decreasing SR specificity as hearing loss increased indicates that middle-aged adults response behavior resembled under-reporting of hearing loss similar to older adults [32]. It is also possible that this result reflects a sample of middle-aged persons from the Midwest and South regions with a different pattern of self reported response behaviors compared to a California sample [25]. The current research method (face-to-face survey) was also different than the California telephone survey which might have influenced participants’ responses to the SR question.

New findings in this study revealed the highest specificity of SR occurred in nonsmokers for mild and moderate hearing losses. One possible reason for this result might be that nonsmokers were pathology free as they reported being healthy, without any effects of nicotine/smoking. Similarly, younger participants followed by nonsmokers had the highest specificity for severe hearing loss and speech scores. This might be due to younger participants having good hearing and health, and being free of aging effects. In support of this explanation, current smokers and middle-aged individuals revealed the lowest specificity in two conditions as discussed earlier.

Overall high specificity in this study suggests that the SR question would be effective in ensuring that fewer people with normal hearing are labeled as hearing impaired in the screening process. This is particularly true for nonsmokers and younger individuals as they had the highest specificity. However, clinical caseloads that include smokers and middle-aged individuals should expect a slightly lower specificity of the SR screening question.

#### Sensitivity

Overall, high sensitivity of the SR question is desirable if the reason for screening adults is to provide intervention. However, the current results do support the benefits associated with quickly and easily screening adults at risk for hearing impairment, such as smokers, and baby boomers who are expected to have been exposed to excessive noise.

In middle-aged participants, sensitivity of the SR question compared with audiometry (mild hearing loss) was lower (46%) than past reports (51-78%) [19, 22, 23, 30]. However, SR sensitivity (90%) improved dramatically with moderate hearing loss and was similar to previous reports (70-93%) [19, 30]. These results suggest that using the SR question alone for hearing screening will successfully identify persons with moderate impairment but possibly miss those with mild hearing loss. In order to ensure that fewer real cases of hearing loss go undetected, such as baby boomers with mild impairment, audiometry should supplement SR screening methods. Given that screening protocol philosophies differ, this recommendation would be supported by ASHA [2] but not by clinicians or researchers who favor a 40 dB criterion for adult hearing screening [32].

Higher sensitivity of the SR question among smokers compared to other participants suggests that SR has a higher probability of correctly identifying hearing loss in smokers. Therefore, clinical use of SR is encouraged for hearing screening in adult smokers. However, the need for supplemental audiometry is evident as discussed above to detect mild impairment in smokers.

#### Predictive Value

The low PPV scores overall are not surprising given the low sensitivity scores. In contrast, NPV scores are excellent across PTA and WRS for all participants. This current result of high NPV and low PPV was reported previously in older individuals [23]. In this study, we reported the same finding for younger individuals, smokers, and nonsmokers. Furthermore, low prevalence rates of moderate and severe hearing loss in middle-aged individuals may partially explain the high NPV and low PPV [23]. Similarly, low prevalence rates of moderate and severe hearing loss in younger individuals and nonsmokers might explain the observed very high NPV and very low or zero PPV.

### Implications for Clinical Practice

The present study revealed excellent sensitivity and specificity of the SR question with a 40 dB (moderate) hearing impairment cutoff. However, sensitivity was sacrificed somewhat with a 25 dB (mild loss) cutoff. These results have implications for nurses and other community health personnel developing adult hearing screening protocols:

- SR screening tools should be considered appropriate for adults, including smokers, baby boomers, and younger individuals.
- Audiometric screening should supplement SR screening to facilitate intervention for adults with mild hearing loss.
- Adults who fail hearing screenings should be referred to an audiologist for diagnostic testing and management of hearing loss.
- Adults with mild hearing losses, especially those not interested in hearing aids, should receive counseling about hearing protection, assistive listening devices, health protection supplements, and avoiding excessive noise and smoking.

### Strengths and Limitations

This study did not seek information about noise exposure history from participants which might be useful to correlate with reported and measured prevalence of hearing loss. Also, data management resulted in the group of smokers including both younger and middle-aged adults. It is possible that baby boomers in the smokers group were potentially impacted by a combination of noise exposure, smoking, and aging effects. Future research might aim to create more homogenous participant groups.

Whereas past studies evaluating self reported and measured hearing loss have focused on older adults, this study included other persons at risk for hearing impairment, including smokers, as well as younger adults and baby boomers possibly exposed to excessive noise. Information from this study with specific suggestions for using SR and audiometry as discussed above would assist public health professionals by guiding screening protocols. In addition, this information may be used to educate physicians, medical residents, and nurses through graduate or continuing education programs. Such clinical and educational efforts would promote hearing health, and support U.S. government mandated Healthy People 2020 initiatives.
